# Performance of visual, manual, and automatic coronary calcium scoring of cardiac ^13^N-ammonia PET/low dose CT

**DOI:** 10.1007/s12350-022-03018-0

**Published:** 2022-06-16

**Authors:** Magdalena M. Dobrolinska, Sergiy V. Lazarenko, Friso M. van der Zant, Lonneke Does, Niels van der Werf, Niek H. J. Prakken, Marcel J. W. Greuter, Riemer H. J. A. Slart, Remco J. J. Knol

**Affiliations:** 1 Medical Imaging Center, Departments of Radiology, Nuclear Medicine and Molecular Imaging, University of Groningen, University Medical Center Groningen, PO Box 30.001, 9700 RB Groningen, The Netherlands; 2 Department of Nuclear Medicine, Northwest Clinics, Alkmaar, The Netherlands; 3 Department of Radiology, University of Utrecht, University Medical Center Utrecht, Heidelberglaan 100, 3584 CX Utrecht, The Netherlands; 4 Department of Radiology & Nuclear Medicine, Erasmus University Medical Center Rotterdam, Postbus 2040, 3000 CA Rotterdam, The Netherlands; 5 Department of Robotics and Mechatronics, Faculty of Electrical Engineering, Mathematics & Computer Science, University of Twente, P.O. Box 217, 7500 AE Enschede, The Netherlands; 6 Department of Biomedical Photonic Imaging, Faculty of Science and Technology, University of Twente, Drienerlolaan 5, 7522 NB Enschede, The Netherlands

**Keywords:** CAD, PET, CT, Image interpretation

## Abstract

**Background:**

Coronary artery calcium is a well-known predictor of major adverse cardiac events and is usually scored manually from dedicated, ECG-triggered calcium scoring CT (CSCT) scans. In clinical practice, a myocardial perfusion PET scan is accompanied by a non-ECG triggered low dose CT (LDCT) scan. In this study, we investigated the accuracy of patients’ cardiovascular risk categorisation based on manual, visual, and automatic AI calcium scoring using the LDCT scan.

**Methods:**

We retrospectively enrolled 213 patients. Each patient received a ^13^N-ammonia PET scan, an LDCT scan, and a CSCT scan as the gold standard. All LDCT and CSCT scans were scored manually, visually, and automatically. For the manual scoring, we used vendor recommended software (Syngo.via, Siemens). For visual scoring a 6-points risk scale was used (0; 1-10; 11-100; 101-400; 401-100; > 1 000 Agatston score). The automatic scoring was performed with deep learning software (Syngo.via, Siemens). All manual and automatic Agatston scores were converted to the 6-point risk scale. Manual CSCT scoring was used as a reference.

**Results:**

The agreement of manual and automatic LDCT scoring with the reference was low [weighted kappa 0.59 (95% CI 0.53-0.65); 0.50 (95% CI 0.44-0.56), respectively], but the agreement of visual LDCT scoring was strong [0.82 (95% CI 0.77-0.86)].

**Conclusions:**

Compared with the gold standard manual CSCT scoring, visual LDCT scoring outperformed manual LDCT and automatic LDCT scoring.

**Supplementary Information:**

The online version contains supplementary material available at 10.1007/s12350-022-03018-0.

## Introduction

Coronary artery calcium (CAC) score is not only a sign of atherosclerotic processes, but also a well-known risk predictor of cardiovascular diseases (CVD) for asymptomatic individuals with an intermediate risk of significant coronary artery stenosis.^1^ A higher CAC score has shown to be associated with a higher risk of atherosclerotic disease.^2,3^ Particularly, individuals with CAC > 100 experience more cardiovascular events, as compared to those with lower CAC scores.^4^ Furthermore, Peng et al showed that the probability of a cardiovascular event even increases when the CAC score exceeds 1 000.^5^ Conversely, the absence of coronary calcium is considered to be the most important negative marker of CVD.^6^ However, the value of CAC scoring is not limited to asymptomatic individuals. Lo‐Kioeng‐Shioe et al demonstrated that CAC scoring also adds value to the prediction of major adverse cardiac events (MACE) in symptomatic patients.^7^

Traditionally, CAC score is calculated from dedicated, ECG-triggered coronary calcium scoring computed tomography (CSCT) scans following the standard manual Agatston scoring method.^8^ The alternatives for time consuming manual calcium scoring are visual and automatic scoring methods. Visual scoring typically categorizes visible CAC by eye balling in one of six groups.^9^ This method has been described in the past decade and is known to have good agreement with the gold standard, CSCT scans.^9^ Recently, new commercially available software has emerged, which employs deep learning methods (DL) to calculate the Agatston score. DL enables automatic calcium scoring, and was previously validated on CSCT scans.^10^

In everyday clinical practice, myocardial perfusion imaging (MPI) positron emission tomography (PET) is preceded by non-ECG triggered low dose CT (LDCT) scans instead of CSCT scans. The LDCTs are used for attenuation correction of the PET data. Importantly, accurate assessment of CAC from LDCT scans would certainly add new information about patients’ risk to the results of MPI. Besides standard non-contrast coronary calcium scoring scans, it was demonstrated that coronary calcium scoring is feasible on almost all diagnostic non-contrast chest CT scans.^11^ As underlined in Society of Cardiovascular Computed Tomography and Society of Thoracic Radiology (SCCT/STR) guidelines, calcium scores derived from LDCT scans should be reported, although there is still insufficient evidence on which method to use.^12^ In this study we therefore decided to use an automatic, clinically available method based on deep learning to measure CAC from LDCT and CSCT scans. In addition, we assessed all LDCT and CSCT scans both visually and manually. The aim of the present study is to compare automatic, manual, and visual coronary calcium scoring performance from LDCT scans acquired during cardiac ^13^N-ammonia PET/CT against manual scoring from dedicated CSCT scans as the gold standard.

## Methods

### Patients

In this single center, retrospective study we included patients who underwent a ^13^N-ammonia-PET/LDCT and a dedicated CSCT scan between 2013 and 2019. All included patients suffered from angina, chest pain, dyspnea, or were suspected of or had known CAD. Each ^13^N-ammonia-PET scan was preceded by CSCT scan, which was typically followed by CCTA. The decision whether or not to proceed with ammonia-PET was made by cardiologist based on CSCT and/or CCTA results, the patient’s symptoms, and patient’s risk group. The time between both scans did not exceed 6 months to minimize any individual changes in calcium scores. Patient exclusion criteria were: myocardial infarction, previous percutaneous coronary intervention (PCI), or PCI between CSCT and ^13^N-ammonia-PET MPI. The study was approved by the local scientific board, and the need to receive approval from the local medical ethical review committee was waived since the study was not within the scope of the Dutch Medical Research Involving Human Subjects Act (section 1.b; February 26, 1998). Additionally, as a standard procedure at the Department of Nuclear Medicine of the Northwest Clinics, all included patients gave written consent to the use of their anonymized data for scientific purposes.

### Data acquisition

#### CSCT protocol

Relevant CSCT data acquisition parameters are presented in Table 1. CSCT scans were prospectively ECG-triggered at 60% of R-R interval without radiocontrast, and during inspiratory breath-hold. A dual source 2 × 64 detector CT system with flying focal spot was used (Somatom Definition Flash, Siemens Healthineers, Forchheim, Germany) at a tube voltage of 120 kVp. The dataset was reconstructed using a B35f medium kernel at 3 mm slice thickness with an increment of 1.5 mm.

**Table 1:** Acquisition and reconstruction parameters for CSCT and LDCT scans

	CSCT	LDCT
Scanner	Somatom Definition Flash, Siemens Healthcare	Biograph TruePoint, Siemens Healthcare
Tube voltage (kVp)	120	130
Qref. mAs	80	25
Field of view	228	500
Collimation (mm)	2 × 64 × 1.2	16 × 1.2
Rotation time (s)	0.285	0.6
Kernel	B35f	B31s
Mode	Spiral	Spiral
Slice thickness	3.0	3.0
Pitch	3.2	0.95
Increment	1.5	3.0
CTDI_vol_ (mGy)	1.3	2.8

#### LDCT protocol

LDCT scans were acquired on a PET/CT system (Biograph-16 TruePoint, Siemens Healthineers, Forchheim, Germany) and performed prior to the ^13^N-ammonia-PET MPI study to serve as attenuation correction CT. LDCT scans were non-ECG-triggered, non-contrast without inspiratory breath-hold. All patients were scanned at 130 kVp. Images were reconstructed with standard filtered back projection using a B31s kernel at 3 mm slice thickness and 1.5 mm increment (Table 1).

### Phantom study

**Figure 1:**
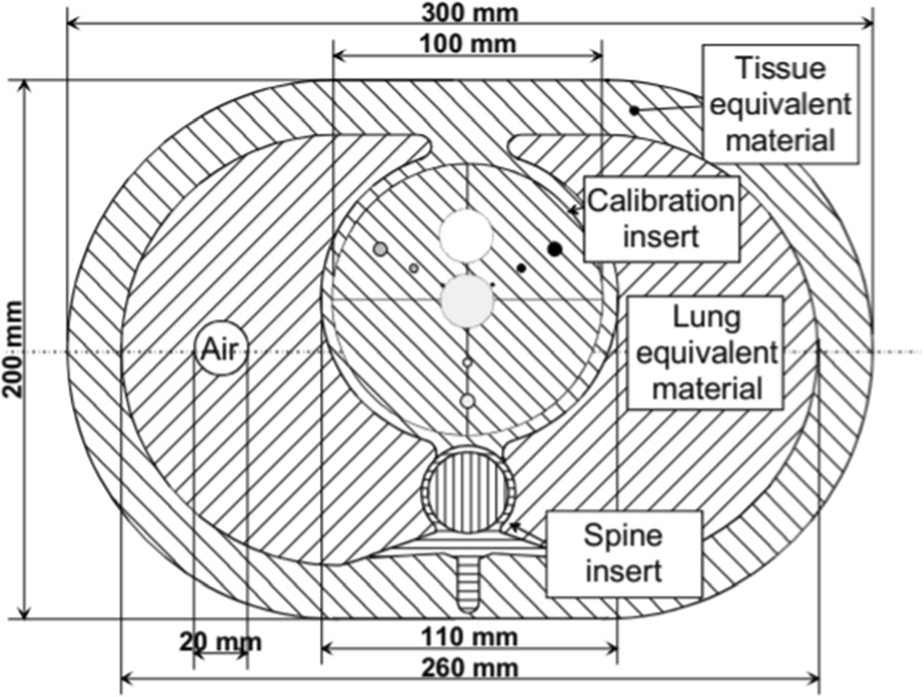
Overview of an anthropomorphic thorax phantom with calcium inserts

In addition, an anthropomorphic thoracic phantom (QRM Thorax phantom, PTW, Germany) with a large calibration insert of hydroxyapatite (200 mg/cm^3^, QRM CCI, PTW, Germany) was scanned with the CSCT and LDCT protocols to determine the calcium detection threshold at 130 kVp (Figure 1), following the method of Thomas et al,^13^ described in the equation below. Each protocol was scanned five times and the mean CT value of the large calibration rod was determined within a large region of interest within the central slice. The recalculated calcium HU threshold played no role in the deep learning algorithm as it was trained and validated on 130 kVp data.^10^

$${t}_{130\mathrm{kVp}}=130 \mathrm{HU} \times \frac{{\mathrm{HU}}_{\mathrm{CaHA}@130\mathrm{kVp}}}{{\mathrm{HU}}_{\mathrm{CaHA}@120\mathrm{kVp}}}$$

*t*_130kVp_ = adapted threshold at 130 kVp, HU_CaHA@130kVp_—HU value of calcium insert scanned at 130 kVp, HU_CaHA@120kVp_—HU value of calcium insert scanned at 120 kVp

### Scoring methods

Both LDCT and CSCT scans were transferred to a workstation (Syngo.via, Siemens Healthineers, Forchheim, Germany) for CAC analysis. All scans were scored visually, manually, and automatically on axial images for each separate artery (LM—left main, LAD—left anterior descending, RCA—right coronary artery, LCx—left circumflex artery) and as a total calcium score. In a per vessel analysis, LM and LAD were taken together as one single vessel.

#### Manual scoring

Manual scoring of CSCT scans was done according to the Agatston method in which calcium is defined by a threshold of 130 HU and an area ≥ 1 mm^2^.^8^ For the manual LDCT scoring, the tube voltage corrected threshold was used. Manual scoring was performed by two observers (L.D. and M.M.D.) using dedicated software (syngo.via CT CaScoring VB50, Siemens Healthineers, Forchheim, Germany).

#### Automatic scoring

The automatic scoring for LDCT and CSCT was performed with a commercially available algorithm, the details of which were explained previously.^10^ In short, the calcium scoring software (syngo.via CT CaScoring VB50, Siemens Healthineers, Forchheim, Germany) uses deep learning methods to determine the calcium score.^10^ It detects calcium containing voxels which exceed the threshold of 130 HU and assigns them to labeled coronary arteries. First, the heart was segmented with a U-Net architecture from the CT volume. Next, the CT volume was cropped to the heart and the coronary map was registered. Finally, a CNN network was applied to mask coronary arteries. As a result, the Agatston score was calculated on a per vessel basis and also as a global Agatston score for the entire coronary tree.^10^

#### Visual scoring

For visual scoring of LDCT and CSCT scans we employed the previously described 6-point patient risk scale (Table 2).^9^ Visual scoring was performed twice by one observer (M.M.D.) blinded to the results of the gold standard CSCT.

**Table 2:** The description of visual 6-point scale which was used for visual calcium scoring

Visual six-point scale	Agatston score equivalent
0	0
1	1–10
2	11–100
3	101–400
4	401–1 000
5	> 1 000

### Statistical analysis

Continuous variables were presented as means (with standard deviations or 95% confidence intervals) or medians (with interquartile range, IQR). Normality of variables was visually assessed based on histograms and q-q plots. Spearman’s correlation was used to calculate correlations between manual and automatic scores. Total and per-vessel manual and automatic methods scores were compared to the gold standard using Bland–Altman plots. For the comparison of non-parametric data, the Wilcoxon signed rank test was used. All manually and automatically measured scores were converted into the six risk groups. The agreement in risk group classification between the different scoring methods was measured using a Cohen weighted linear *κ* with 95% confidence intervals (95% CI). The kappa coefficients were categorized as: 0.01-0.2: slight agreement, 0.21-0.4: fair agreement, 0.41-0.6: moderate agreement, 0.61-0.8: substantial agreement, and 0.81-0.99 excellent agreement.^14^ An Agatston score of ≥ 1 was defined as CAC positive. The sensitivity, specificity, positive predictive value (PPV) and negative predictive value (NPV) of CAC detection on LDCT scans was calculated.^15^ A *P* value < 0.05 was considered statistically significant. Statistical analyses were performed with Statistical Package for the Social Sciences (SPSS v 23; IBM, Armonk, NY) and MedCalc (MedCalc 15.8, MedCalc Software).

## Results

### Phantom results

The average CT-value of the calibration insert was 249 and 269 HU, at 130 kVp and 120 kVp, respectively. The calcium HU threshold for a tube voltage of 130 kVp was calculated at 123 HU.

### Patients’ characteristics

In total, 213 patients met the inclusion criteria, 111 (52.4%) were men. Mean patients’ age was 64 ± 9 years. Median time between LDCT and CSCT scans was 4 (2.0, 4.0) weeks. The available clinical information of 174 out of 213 study participants is summarized in Table 3. Agatston score results from CSCT scans are shown in Table 4.

**Table 3:** Baseline characteristics of study participants, myocardial

	Total	Male	Female
	n = 174/213	54.6%	45.4%
Age (y)	61.7 ± 9.3	60.4 ± 8.8	63.1 ± 9.7
BMI	27.9 ± 4.4	27.3 ± 4.1	28.7 ± 4.7
Risk factors			
Positive family history (%)	44.4	39.1	50.6
Smoking (%)	17	15.2	18.9
Diabetes (%)*	9.4	12.0	6.3
Hypercholesterolemia (%)	39.2	35.9	43.0
Hypertension (%)	51.5	41.3	63.3
Previous cardiac events			
Previous MI (%)	0.6	1.1	0
Previous PCI (%)	2.3	3.3	1.3
Previous CABG (%)	0.6	1.1	0
Duke Clinical Score^28^	40.0 ± 26.1	52.6 ± 24.4	26.5 ± 20.7
Cardiac medication			
None (%)	10.2	8.9	11.7
Statins (%)	71.9	73.3	70.1
Anticoagulants (%)	65.9	70.0	61.0
Betablocker (%)	55.7	54.4	57.1
Calcium antagonist (%)	16.8	21.1	11.7
AT2-antagonist or ACE-inhibitor (%)	32.9	31.1	35.1
Diuretics (%)	12.0	10.0	14.3
Nitrates (%)	5.4	5.6	5.2

Table summarizes clinical information about available 174 out of 213 patients. Values are presented as% or means (± SD) of 174/213 patients, due to limited availability.

*CABG* coronary artery bypass grafting; *MI* myocardial infarction; *PCI* percutaneous coronary intervention

*Diabetes Type I and Type II

**Table 4:** Results of baseline CSCT scan

	Baseline characteristics
CAC categories based on Agatston score from CSCT scans	
0	12 (5.6%)
1	9 (4.2%)
2	27 (12.7%)
3	34 (16.0%)
4	69 (32.4)
5	62 (29.1)
Agatston score from CSCT scans	
CAC total	579.4 (139.4, 1103.85)
CAC LM	0.0 (0.0, 30.5)
CAC LAD	269.4 (73.2, 465.95)
CAC LCx	40.2 (0.45, 192.65)
CAC RCA	97.3 (1.1, 428.85)

Values are presented as n (%).

*CAC* coronary artery calcium; *CSCT* coronary calcium score CT scan; *LAD* left anterior descending; *LCx* left circumflex artery; *LM* left main; *RCA* right coronary artery

### Automatic, visual, and manual scoring of CAC from CSCT scans

#### CSCT calcium score analysis

**Figure 2:**
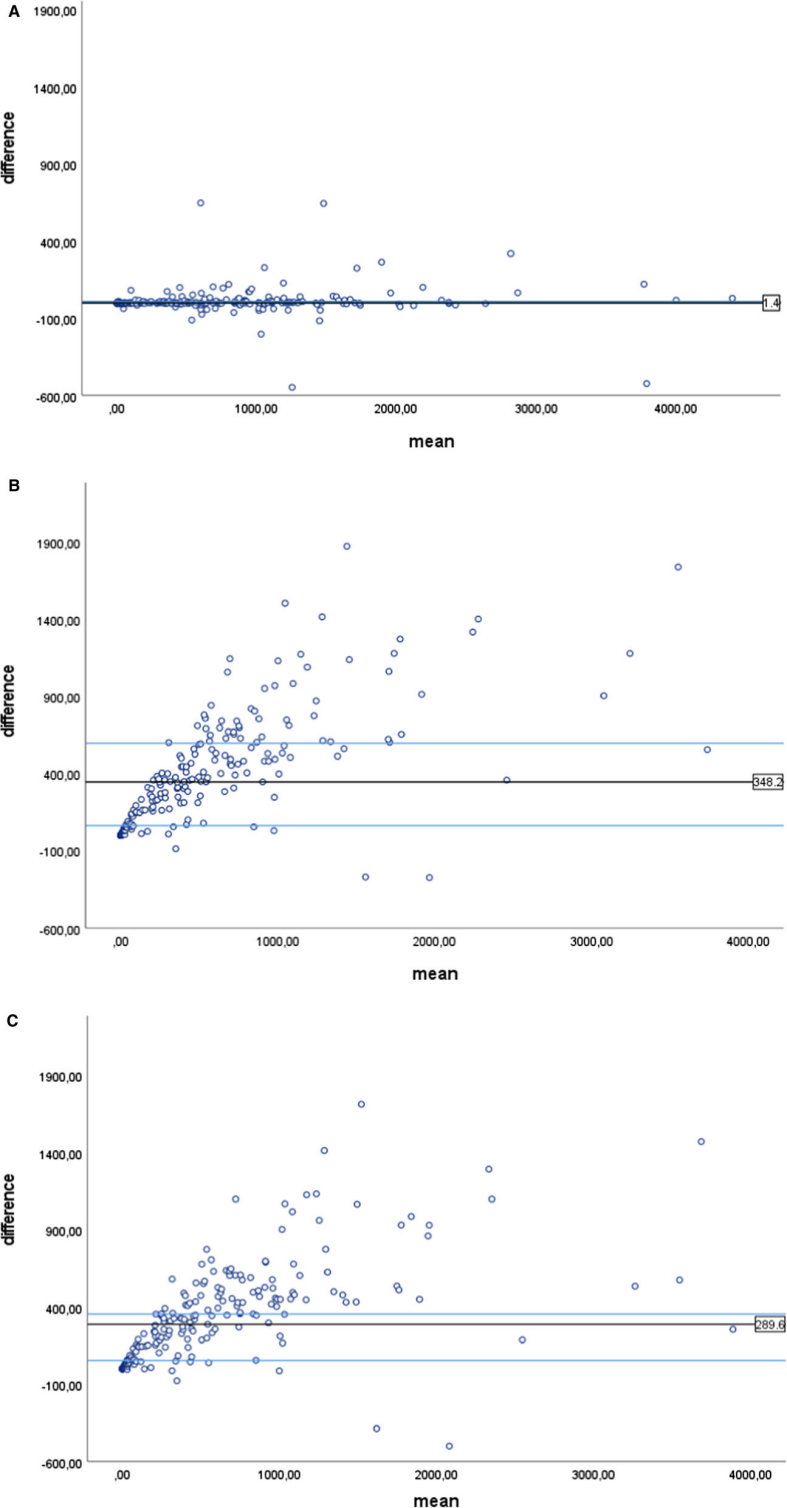
Bland–Altman plots showing the median difference between Agatston score measured manually from CSCT scans and (**A**) Agatston score measured automatically from CSCT scans, (**B**) Agatston score measured automatically from LDCT scans, (**C**) Agatston score measured manually from LDCT scans

*Total manual agatston score vs automatic scoring* The median value of total Agatston score was similar for the manual and automatic scoring methods: 579.4 (IQR 139.4, 1103.8) and 589.9 (IQR 129.1, 1100.3), respectively. The median difference between manual and automatic Agatston score measured from CSCT scans was 1.4 (95% CI − 0.1-11.45) (Figure 2A). There was an excellent correlation between manual and automatic methods (*r* = 0.99; *P* < .001). The agreement between manual and automatic Agatston score risk group classification was excellent (*κ* = 0.95, 95% CI 0.92-0.97) (Table 5, Supplementary Table S1). 91% scans were assigned to the same category. Based on manual scoring from CSCT scan, 5.6% of the included patients had an Agatston score of zero. Based on the automatic method, 0.9% of scans was incorrectly assigned to the zero Agatston score group (Table 5, Supplementary Table S1).

**Table 5:** The agreement between automatic and visual scoring from CSCT scans and manual, automatic, and visual scoring from LDCT scans with a gold standard

	CSCT		LDCT		
	Automatic	Visual	Manual	Automatic	Visual
Weighted linear *κ*	0.95	0.88	0.59	0.50	0.82
95% CI	0.92–0.97	0.85–0.92	0.53–0.65	0.44–0.56	0.77–0.87
% of cases within the same category	91.0	82.1	35.2	29.5	74.2
% of cases within 1 category below or above the correct one	100	100	94.3	93.6	98.1
% of cases incorrectly assigned as 0 Agatston score	0.9	0	4.2	12.7	3.2

#### Total manual Agatston score vs visual scoring

The agreement of risk group classification between manual and visual Agatston score was excellent (*κ* = 0.88, 95% CI 0.85-0.92). 82.1% of scans were within the same category. Based on visual analysis, none of the scans was misclassified into the zero Agatston score group (Table 5).

### Automatic, visual, and manual scoring of CAC from LDCT scans

#### LDCT calcium score analysis

*Automatic assessment from LDCT vs gold standard* The total Agatston score automatically derived from LDCT scans was significantly lower compared to that of CSCT scans (206.9 (IQR 20.5, 492.1) vs. 579.4 (IQR 139.4, 1103.8); *P* < .001). Correlation between two scores was excellent (*r* = 0.93; 0 < 0.001). The median difference between the automatically derived Agatston scores from LDCT as compared to CSCT scans in the per-patient analysis was 348.2 (IQR 64.45, 597.6) (Figure 2B). Based on the per vessel analysis, the highest variations in calcium score results were found in LM-LAD (99.7, IQR 15.25, 234.75, Supplementary Table S2). The agreement (*κ*) between the results of the automatic Agatston scoring method in both CSCT and LDCT scans versus the gold standard was only 0.5 (95% CI 0.44-0.56). 29% of cases were assigned to the same risk category, and 93.6% of cases fell within one risk category (one risk category below or above the correct one). Using the automatic analysis method, 12.7% of patients were incorrectly assigned to the zero Agatston score category (Tables 5, 6B). The specificity, sensitivity, PPV and NPV were 100%, 81.7%, 100.0%, and 30.8%, respectively (Table 7).

**Table 6:** Agreement in risk classification between (A) automatic, (B) manual and (c) visual assessment of LDCT scans and gold standard.

(A)							
Automatic LDCT	Agatston score measured on CSCT						
	0	1–10	11–100	101–400	400–1 000	> 1 000	
0	12	9	14	4	0	0	39 (18.3%)
1–10	0	0	7	0	1	0	8 (3.8%)
11–100	0	0	6	16	7	0	29 (13.6%)
101–400	0	0	0	14	49	9	72 (33.8%)
401–1 000	0	0	0	0	12	34	46 (21.6%)
> 1 000	0	0	0	0	0	19	19 (8.9%)
	12 (5.6%)	9 (4.2%)	27 (12.7%)	34 (16.0%)	69 (32.4%)	62 (29.1%)	213

(B)							
Manual LDCT	Agatston score measured on CSCT						
	0	1–10	11–100	101–400	400–1 000	> 1 000	
0	12	7	2	0	0	0	21 (9.9%)
1–10	0	2	13	2	0	0	17 (8.0%)
11–100	0	0	12	18	4	0	34 (16.0%)
101-400	0	0	0	14	49	4	67 (31.5%)
401–1 000	0	0	0	0	16	39	55 (25.8%)
> 1 000	0	0	0	0	0	19	19 (8.9%)
	12 (5.6%)	9 (4.2%)	27 (12.7%)	34 (16.0%)	69 (32.4%)	62 (29.1%)	213

(C)							
Visual LDCT	Agatston score measured on CSCT						
	0	1–10	11–100	101–400	400–1 000	> 1 000	
0	12	5	1	1	0	0	19 (8.9%)
1–10	0	4	4	1	0	0	9 (4.2%)
11–100	0	0	20	8	0	0	28 (13.1%)
101–400	0	0	2	22	16	1	41 (19.2%)
401–1 000	0	0	0	2	53	14	69 (32.4%)
> 1 000	0	0	0	0	0	47	47 (22.1%)
	12 (5.6%)	9 (4.2%)	27 (12.7%)	34 (16.0%)	69 (32.4%)	62 (29.1%)	213

A: Weighted linear *κ* = 0.50 (95% CI 0.44–0.56)

B: Weighted linear *κ* = 0.58 (95% CI 0.52–0.63)

C: Weighted linear *κ* = 0.82 (95% CI 0.77–0.87)

**Table 7:** Sensitivity, specificity, PPV and NPV of CAC detectability on LDCT scans as compared to gold standard

	Manual LDCT	Automatic LDCT	Visual LDCT
Sensitivity	95.5	81.7	96.5
Specificity	100.0	100.0	100.0
PPV	100.0	100.0	100.0
NPV	57.1	30.8	63.2

*LDCT* low dose CT scan; *NPV* negative predictive value; *PPV* positive predictive value

*Manual assessment from LDCT scans vs gold standard CSCT* The total manually measured Agatston score on LDCT scans was significantly lower compared to CSCT scans (247.1 (IQR 32.4, 578.8) vs. 579.4 (IQR 139.4, 1103.8); *P* < .001). The median difference between total Agatston scores in the per-patient analysis was 289.6 (IQR 55.5, 493.30) (Figure 2C). Similar to the automatic scoring method, the highest variation was found in the LM-LAD, in the per vessel analysis (99.9, IQR 16.8, 217.95, Supplementary Table S2). The agreement (κ) of calcium risk group analysis between the gold standard and the manual total Agatston scoring on LDCT scans was 0.58 (95% CI 0.52-0.63). 4.2% of cases were incorrectly assigned to the zero Agatston score category (Tables 5, 6B). The specificity, sensitivity, PPV and NPV were 100%, 95.5%, 100.0%, and 51.7%, respectively (Table 7). The inter-observer agreement on manual LDCT calcium scoring is summarized in Supplementary Table S3.

*Visual assessment of LDCT vs gold standard CSCT* Agreement (*κ*) between visual scoring based on LDCT scans and the gold standard was 0.82 (95% CI 0.77–0.87). Compared to the gold standard, 74.2% of cases were assigned to the same category and 98.1% fell within one category (one risk category below or above the correct one). As compared to the automatic and manual method, the lowest number of cases were incorrectly assigned to the zero Agatston score category (3.2%) (Tables 6c, 7). Of the three evaluated calcium scoring methods from LDCT scans, visual scoring had the highest sensitivity and NPV (96.5%, 63.2%, respectively). The intra-observer agreement of visual calcium scoring from LDCT scans was high (*κ* = 0.94, 95% CI 0.92-0.96) and is summarized in Supplementary Table S4.

## Discussion

The present study provides information about the applicability of a newly developed, clinically available, AI powered calcium scoring method, and visual assessment and traditional manual calcium scoring techniques using LDCT scans, compared to the results of the gold standard—manual calcium scoring on dedicated CSCT datasets. The results indicate that all three scoring methods correctly identify patients with CAC, as reflected in the high positive predictive values. Nevertheless, none of the scoring methods reliably excludes the presence of calcification, as reflected in the low negative predictive value. Visual calcium LDCT scoring provided the highest agreement with manual CSCT scoring.

### AI in calcium scoring from LDCT scans

A large and growing body of literature has assessed different methods of calcium scoring from LDCT scans. This is, to our knowledge, the first study which implements a new, automatic, commercially available AI powered calcium scoring technique on LDCT scans.^10^ In addition to automatic scoring, we employed manual and visual scoring, and compared the results to the gold standard CSCT assessment. As a first step, we applied the automatic method on CSCT scans, which resulted in a comparable agreement in risk group classification as reported by Winkel et al (*κ* 0.95 vs 0.89, respectively).^10^ For LDCT calcium scores, however, the agreement dropped to 0.5. Despite this low agreement in risk group classification, in 93% of the scans the risk reclassification did not vary by more than one risk group. Moreover, the high specificity and positive predictive value of the automatic method indicated a correct identification of patients with CAC. Other studies in which automatic methods were applied to both CSCT scans and non-gated LDCT scans, outperformed the method we applied in our study. Recently, Zeleznik et al presented a deep learning method of calcium scoring which was applied on both gated and non-gated scans, with an overall agreement of 0.7.^16^ Additionally, a fully automated CAC scoring method presented by Isgum et al demonstrated an agreement of 0.74 between LDCT scans and the gold standard.^17^ The measurements performed by the automatic algorithm of Isgum et al were done on ECG-gated scans, using a DL algorithm that was trained for such gated scans. In contrast, the automatic method used in our study was not trained on non-gated scans.^10^ Lack of ECG-triggering increases the amount of motion artifacts, decreases the accuracy of calcium detection and hence, potentially hampers quantification,^18^ especially when the DL algorithm was not trained on this type of data.^19^ This may explain the lower agreement with the gold standard, as compared to the abovementioned studies.

It is interesting to note that in our study both automatic and manual calcium scoring from LDCT scans significantly underestimated the Agatston score. One explanation for this is that motion artifacts influence the number of voxels exceeding the 130 HU threshold.^18^ In studies performed by Kaster et al and Mylonas et al, the calcium scoring threshold has been changed as low as 50 HU.^20,21^ It should be underlined that as the HU threshold decreases, the false positive results increase due to higher noise levels. Moreover, the resulting calcium score is no longer an Agatston score by definition.^8^ As reported by Mylonas et al, the highest agreement with the gold standard was achieved for a calcium threshold of 50 HU.^21^ Nevertheless, these findings were not repeated elsewhere, and the value of the threshold was based on a very small sample size. Taking together, in our study the correlation between manual and automatic LDCT scoring as compared to the gold standard method was excellent. Nevertheless, systematic underestimation of the Agatston score resulted in a low overall agreement in risk classification.

Much of the current literature which focusses on automatic calcium assessment from LDCT scans highlights automatic methods of Agatston scoring. However, the lack of one, commonly used, validated protocol for LDCT scans, limits the application of Agatston scoring, which is a strictly defined method for calcium measurement.^8^ Additionally, the majority of literature focusing on automatic methods, does not include the gold standard as a comparison. This may generally overestimate the performance of AI methods in calcium scoring.

### Visual calcium scoring from LDCT scans

A visual analysis of calcium score was previously introduced by Einstein et al.^9^ This simple method, repeated by others, has demonstrated good agreement with the gold standard.^22–24^ In our study, of all applied methods, visual assessment of LDCT scan gained the highest agreement with CSCT calcium scoring. This is in line with the study of Einstein et al, who reported that 63% of visually estimated scores falls into the same category, while Engbers et al reported 71%.^9,22^ In our study, 74.2% cases were correctly assigned to the same category and 94% did not vary by more than one risk category. Moreover, as compared to manual and automatic method, visual analysis yielded high sensitivity and good negative predictive value, which enables high-risk patients’ detection.

### Comparison of patients’ risk groups

The number of risk groups used in various studies complicates direct comparison between studies. For instance Zeleznik et al applied four risk groups, while the group of Isgum used a five risk group classification.^16,17^ In our study, we decided to apply a six-risk group classification, which hampers a direct comparison with studies such as those by Zeleznik and Isgum. Our choice was justified by the fact that we aimed to evaluate how effective LDCT might be in the detection of high-risk group patients with an Agatston score > 1 000. Both automatic and manual assessment detected 19 out of 62 (30.6%) patients from the highest risk group. In terms of high-risk patient detection, visual analysis outperformed other techniques, correctly defining 47 out of 62 (75.8%) patients, which is comparable to the analysis conducted by Einstein et al Importantly, both groups of Einstein and Engbers, used a six-point risk scale, which enables a comparison of the results with our study.^9,22^

### Clinical implications

According to Blaha et al, a coronary artery calcium score of zero is the most important negative risk predictor in asymptomatic and symptomatic patients.^6,25^ Therefore, the greatest concern with LDCT scans is the underestimation of coronary calcium due to inability to detect small calcifications. In our study, low-risk patients were the most challenging group of patients to be identified, and this is reflected in a low sensitivity and negative predictive value of these tests. That was mostly pronounced in automatic scoring of LDCT, when 12.7% of patients were misclassified as zero Agatston score. Based on visual analysis, 3.2% of patients was misclassified as zero Agatston score despite having calcium on CSCT scan. This is lower than reported by the group of Einstein (22%), which might be explained by a relatively low amount of zero Agatston score scans in our study as compared to Einstein et al (5.6% vs 71.1%, respectively).^9^

Notwithstanding the clinical value of PET myocardial perfusion imaging, this method may underestimate the importance of the disease in patients with non-flow limiting coronary artery atherosclerosis, by leaving the incorrect impression of ‘being healthy’. The additional information from LDCT scans about calcium signalizes the presence of atherosclerotic disease, which changes further patient management.^23^ As already noticed and underlined by the Society of Cardiovascular Computed Tomography and Society of Thoracic Radiology, CAC should be reported even when found on non-contrast chest CT scans, however the optimal method of scoring is still not defined.^11^ Based on our analysis, the visual scoring, which is a time-efficient method, demonstrated a good agreement with gold standard, and as shown by Engbers et al, and Patchett et al, may add a clinical value to MPI-PET scan.^23,26^

### Study limitations

This study has some limitations. First of all, LDCT scans were non-ECG triggered scans, characterized by a number of motion artifacts, which are a classic problem of these scans and significantly influences calcium measurement. Secondly, the study was performed using a relatively small sample size and further investigation is needed to confirm our results. Furthermore, patients were repositioned between CSCT and LDCT scans, and this might also account for discrepancy between results.^27^ Additionally, the clinical AI algorithm we applied was not yet optimized for non-gated CT scans. Moreover, it was a single center study and all scans were acquired with the same protocol and identical scanners. On one hand this helped to unify the results and to draw conclusions, on the other hand the overall performance as compared with other scanning protocols and with different vendors remains unknown.

## Conclusions

In conclusion, visual calcium scoring from LDCT scans outperformed manual and automatic analysis and demonstrated the highest agreement with the reference CSCT. Within all three methods, automatic scoring gained the lowest sensitivity and NPV in calcium detectability. Nevertheless, each of abovementioned methods correctly defined patients with CAC. These results provide further support for the statement that CAC can be reported from LDCT scans, with visual scoring to be the most reliable method.

## New knowledge gained

Visual assessment of calcium scores on LDCT scans outperforms both deep learning assisted and classic manual scoring methods and shows the best agreement with reference measurements on dedicated, ECG-triggered CSCT scans in the same patient.

## Supplementary Information

Below is the link to the electronic supplementary material.

Supplementary file1 (DOCX 19 kb)

Supplementary file2 (PPTX 6811 kb)

## Abbreviations

CAC: Coronary artery calcium
CSCT: Calcium scoring CT scan
LDCT: Low dose CT scan
AI: Artificial Intelligence
