# Supplementary material for: Performance of visual, manual, and automatic coronary calcium scoring of cardiac 13N-ammonia PET/low dose CT
Source: J Nucl Cardiol. 2022 Jun 16;30(1):239–50. doi: 10.1007/s12350-022-03018-0 (PMC9984321; doi:10.1007/s12350-022-03018-0)
Supplement: Supplementary file 1 — Supplementary file1 (DOCX 19 kb) [file 12350_2022_3018_MOESM1_ESM.docx]

Supplementary material

**Table S1 Agreement between automatic measurement of Agatston score from CSCT scans and Agatston score measured on CSCT scans, both converted into 6-point scale.**

|  | Agatston score measured on CSCT | | | | | |  |
| --- | --- | --- | --- | --- | --- | --- | --- |
| Automatic CSCT | 0 | 1-10 | 11-100 | 101-400 | 400-1000 | >1000 |  |
| 0 | 10 | 2 | 0 | 0 | 0 | 0 | 12 (5,6%) |
| 1 - 10 | 2 | 7 | 2 | 0 | 0 | 0 | 11 (5,2%) |
| 11-100 | 0 | 0 | 25 | 2 | 0 | 0 | 27 (12,7%) |
| 101-400 | 0 | 0 | 0 | 32 | 3 | 0 | 35 (16,4%) |
| 401 - 1000 | 0 | 0 | 0 | 0 | 62 | 2 | 64 (30,0%) |
| >1000 | 0 | 0 | 0 | 0 | 4 | 60 | 64 (30,0%) |
|  | 12 (5,6%) | 9(4,2%) | 27 (12,7%) | 34 (16,0%) | 69 (32,4%) | 62(29,1%) | 213 |

**Weighted linear κ = 0.95 (95%CI 0.92 – 0.97)**

**Table S2 The summary of per vessel analysis.**

|  | Left circumflex artery | | | Right coronary artery | | | Left main and left anterior  descending coronary arteries | | |
| --- | --- | --- | --- | --- | --- | --- | --- | --- | --- |
|  | Gold standard | LDCT manual | LDCT automatic | Gold standard | LDCT manual | LDCT automatic | Gold  standard | LDCT manual | LDCT automatic |
| Median | 40,2 | 1,9* | 0,0* | 97,3 | 21,0* | 2,9* | 288,8 | 127,8* | 116,3* |
| IQR | 0,4-192,6 | 0,0-2,4 | 0,0-46,2 | 1,1-428,8 | 0,0-52,1 | 0,0-96,3 | 77,5-497,1 | 12,4-308,5 | 7,15-300,8 |

***- indicates significant difference as compared to gold standard (p < 0.05)**

**Gold standard –manual scoring of coronary calcium CT scan**

**LDCT – low dose CT scan**

**Table S3 Agreement between reader 1 and reader 2 in manual calcium scoring on LDCT scans**

|  |  | Observer 1 | | | | | |  |
| --- | --- | --- | --- | --- | --- | --- | --- | --- |
|  |  | 0 | 1-10 | 11-100 | 101-400 | 400-1000 | >1000 |  |
| Observer 2 | 0 | 21 | 0 | 0 | 0 | 0 | 0 | 21(9.9%) |
|  | 1 - 10 | 0 | 16 | 1 | 0 | 0 | 0 | 17(8.0%) |
|  | 11-100 | 0 | 4 | 28 | 2 | 0 | 0 | 34(16.0%) |
|  | 101-400 | 0 | 0 | 3 | 61 | 3 | 0 | 67(31.5%) |
|  | 401-1000 | 0 | 0 | 0 | 5 | 50 | 0 | 55(25.8%) |
|  | >1000 | 0 | 0 | 0 | 0 | 0 | 19 | 19(8.9%) |
|  |  | 21(9.9%) | 20(9.4%) | 32(15.0%) | 68(31.9%) | 53(24.9%) | 19(8.9%) | 213 |

**Weighted linear κ = 0.94 (95 % CI: 0.92 – 0.97)**

**Table S4 Intra-observer agreement in visual calcium scoring on LDCT scans**

|  |  | | First measurement | | | | | | | |  |
| --- | --- | --- | --- | --- | --- | --- | --- | --- | --- | --- | --- |
|  |  | | 0 | 1-10 | 11-100 | | | 101-400 | 400-1000 | >1000 |  |
| Second measurement | 0 | 17 | | 1 | | 0 | 0 | | 0 | 0 | 18 (8.5%) |
|  | 1 - 10 | 1 | | 6 | | 2 | 0 | | 0 | 0 | 9 (4.2%) |
|  | 11-100 | 0 | | 2 | | 19 | 3 | | 0 | 0 | 24 (11.3%) |
|  | 101-400 | 1 | | 0 | | 7 | 28 | | 12 | 0 | 48 (22.5%) |
|  | 401-1000 | 0 | | 0 | | 0 | 10 | | 53 | 7 | 70(32.9%) |
|  | >1000 | 0 | | 0 | | 0 | 0 | | 4 | 40 | 44(20.7%) |
|  |  | 19(8.9%) | | 9(4.2%) | | 28(13.1%) | 41(19.2%) | | 69(32.4%) | 47(22.1%) | 213 |

**Weighted linear κ = 0.94 (95 % CI: 0.92 – 0.96)**
