# Supplementary material for: Performance of visual, manual, and automatic coronary calcium scoring of cardiac 13N-ammonia PET/low dose CT
Source: J Nucl Cardiol. 2022 Jun 16;30(1):239–50. doi: 10.1007/s12350-022-03018-0 (PMC9984321; doi:10.1007/s12350-022-03018-0)
Supplement: Supplementary file 2 — Supplementary file2 (PPTX 6811 kb) [file 12350_2022_3018_MOESM2_ESM.pptx]

## Slide 1
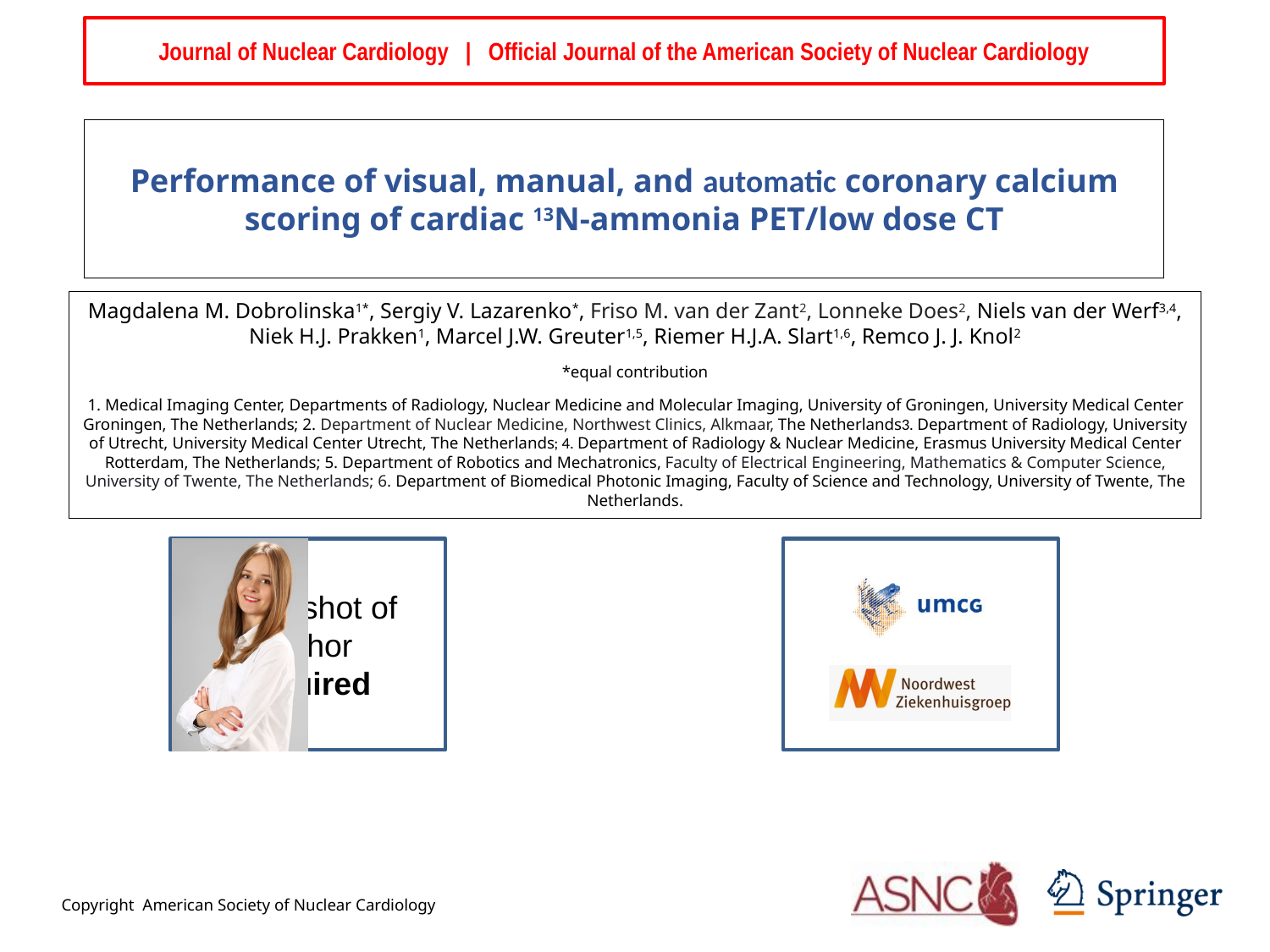

Journal of Nuclear Cardiology | Official Journal of the American Society of Nuclear Cardiology
# Performance of visual, manual, and automatic coronary calcium scoring of cardiac 13N-ammonia PET/low dose CT
Magdalena M. Dobrolinska1*, Sergiy V. Lazarenko*, Friso M. van der Zant2, Lonneke Does2, Niels van der Werf3,4, Niek H.J. Prakken1, Marcel J.W. Greuter1,5, Riemer H.J.A. Slart1,6, Remco J. J. Knol2
*equal contribution
1. Medical Imaging Center, Departments of Radiology, Nuclear Medicine and Molecular Imaging, University of Groningen, University Medical Center Groningen, The Netherlands; 2. Department of Nuclear Medicine, Northwest Clinics, Alkmaar, The Netherlands3. Department of Radiology, University of Utrecht, University Medical Center Utrecht, The Netherlands; 4. Department of Radiology & Nuclear Medicine, Erasmus University Medical Center Rotterdam, The Netherlands; 5. Department of Robotics and Mechatronics, Faculty of Electrical Engineering, Mathematics & Computer Science, University of Twente, The Netherlands; 6. Department of Biomedical Photonic Imaging, Faculty of Science and Technology, University of Twente, The Netherlands.
Head shot of author
required
Copyright American Society of Nuclear Cardiology

## Slide 2
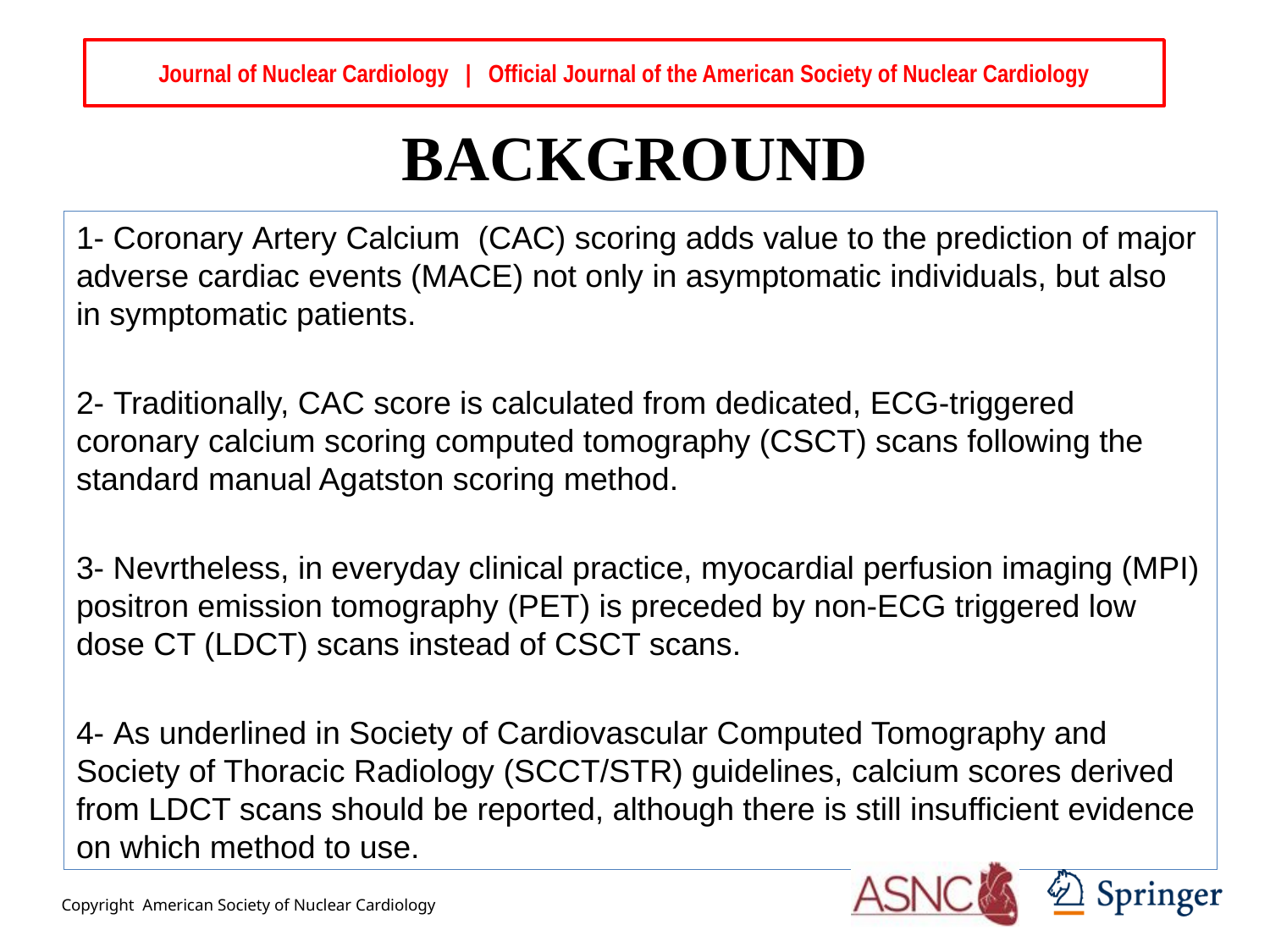

Journal of Nuclear Cardiology | Official Journal of the American Society of Nuclear Cardiology
# BACKGROUND
1- Coronary Artery Calcium (CAC) scoring adds value to the prediction of major adverse cardiac events (MACE) not only in asymptomatic individuals, but also in symptomatic patients.
2- Traditionally, CAC score is calculated from dedicated, ECG-triggered coronary calcium scoring computed tomography (CSCT) scans following the standard manual Agatston scoring method.
3- Nevrtheless, in everyday clinical practice, myocardial perfusion imaging (MPI) positron emission tomography (PET) is preceded by non-ECG triggered low dose CT (LDCT) scans instead of CSCT scans.
4- As underlined in Society of Cardiovascular Computed Tomography and Society of Thoracic Radiology (SCCT/STR) guidelines, calcium scores derived from LDCT scans should be reported, although there is still insufficient evidence on which method to use.
Copyright American Society of Nuclear Cardiology

## Slide 3
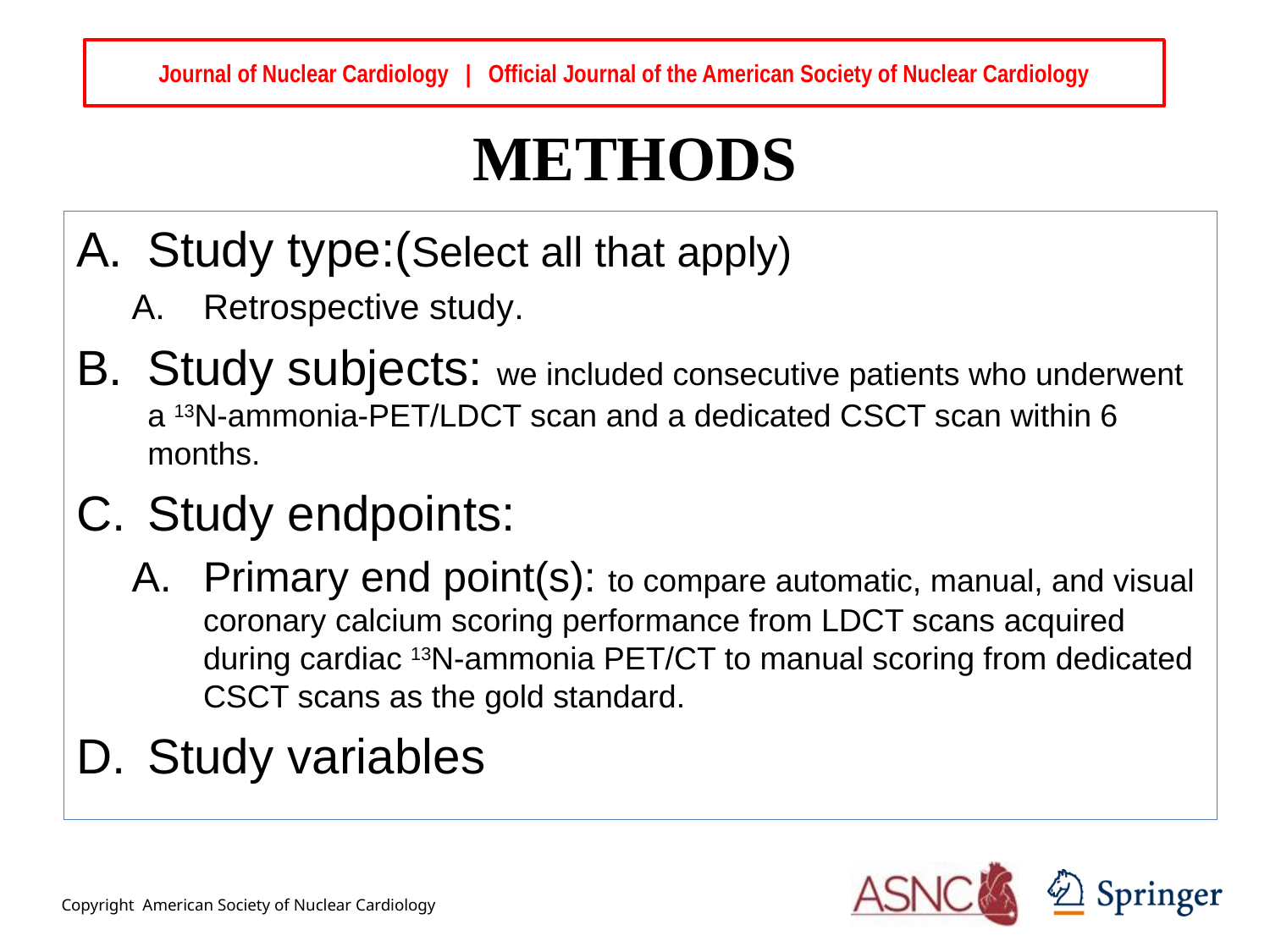

Journal of Nuclear Cardiology | Official Journal of the American Society of Nuclear Cardiology
# METHODS
Study type:(Select all that apply)
Retrospective study.
Study subjects: we included consecutive patients who underwent a 13N-ammonia-PET/LDCT scan and a dedicated CSCT scan within 6 months.
Study endpoints:
Primary end point(s): to compare automatic, manual, and visual coronary calcium scoring performance from LDCT scans acquired during cardiac 13N-ammonia PET/CT to manual scoring from dedicated CSCT scans as the gold standard.
Study variables
Copyright American Society of Nuclear Cardiology

## Slide 4
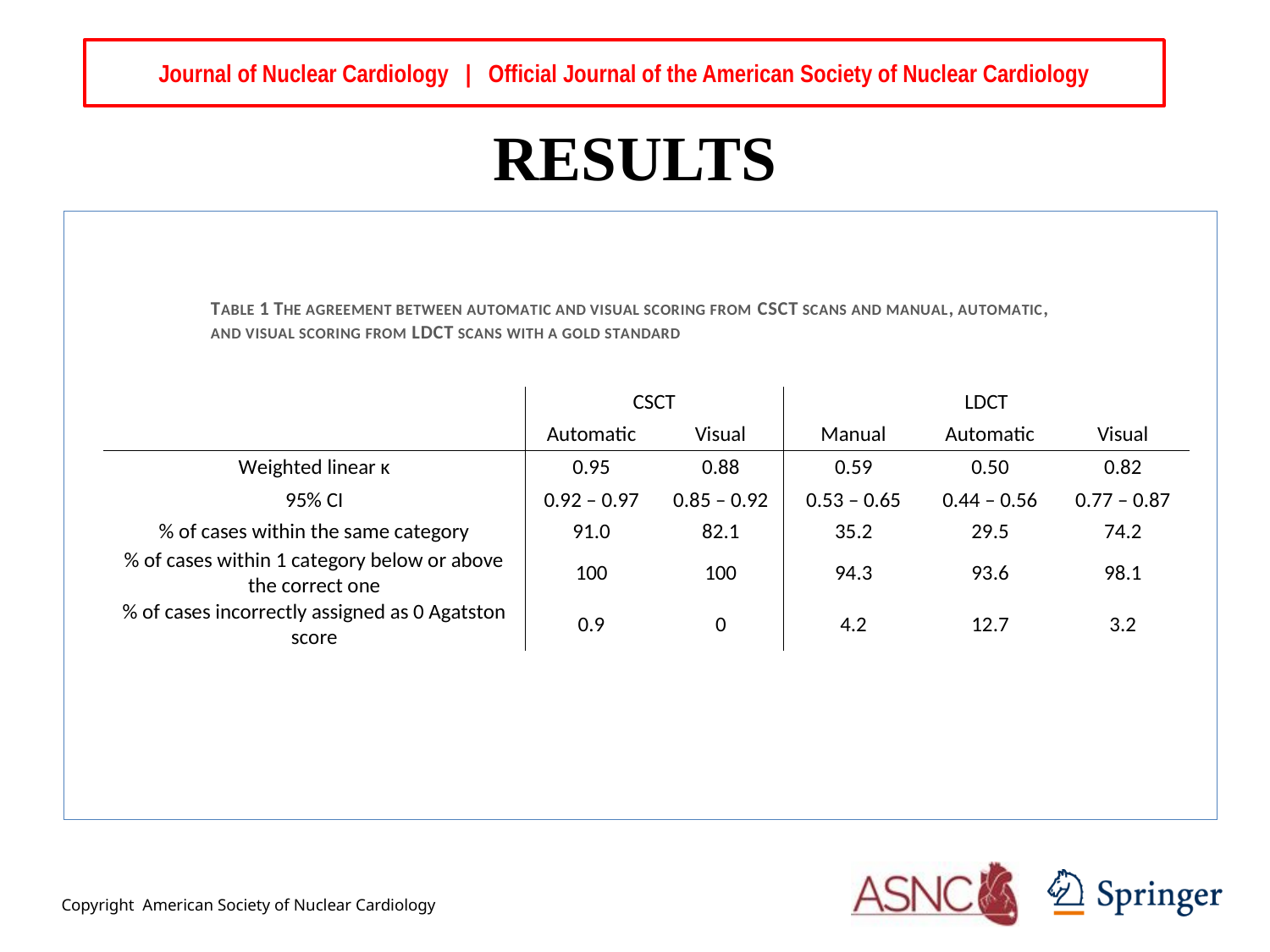

Journal of Nuclear Cardiology | Official Journal of the American Society of Nuclear Cardiology
# RESULTS
Copyright American Society of Nuclear Cardiology

## Slide 5
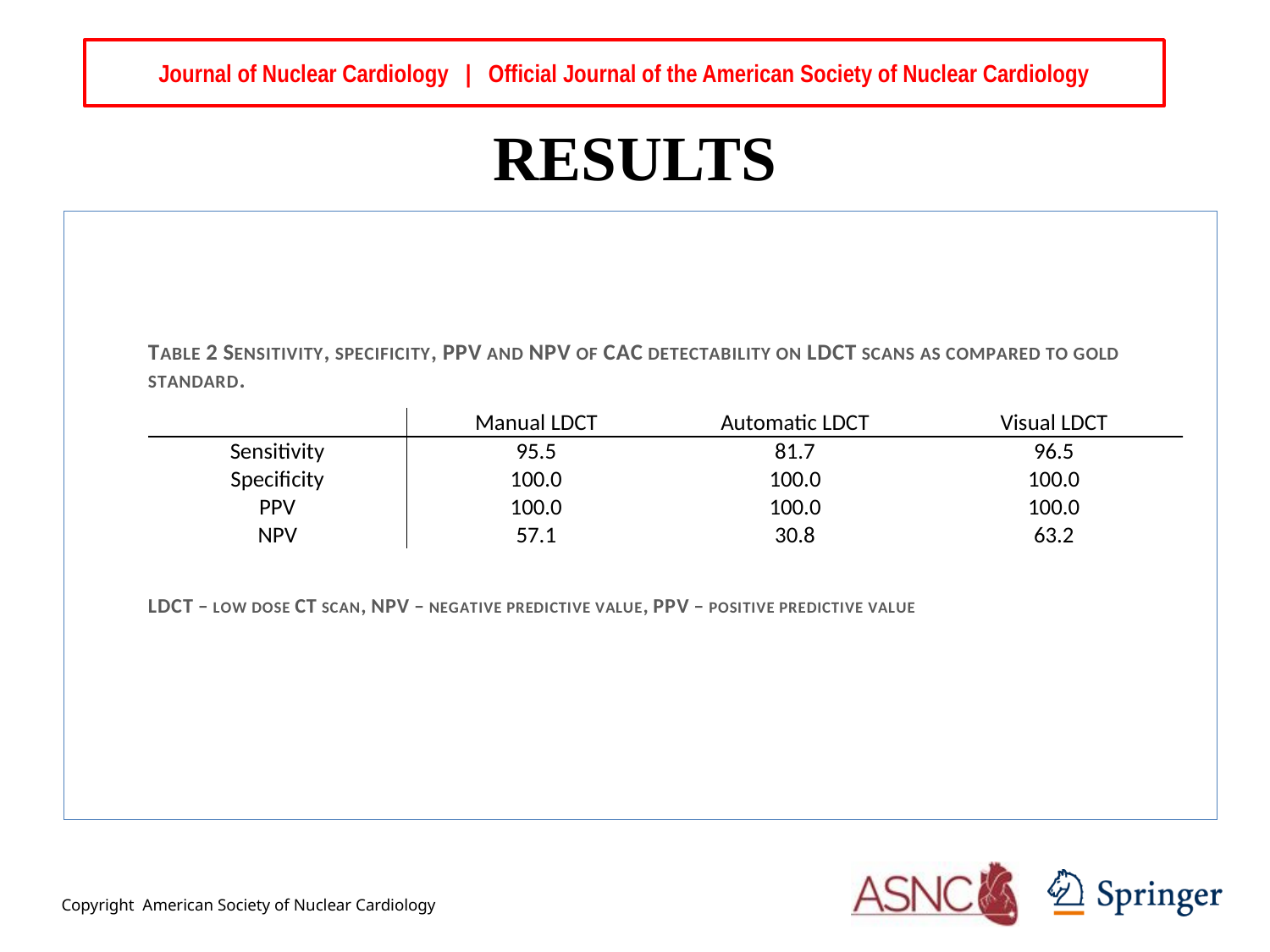

Journal of Nuclear Cardiology | Official Journal of the American Society of Nuclear Cardiology
# RESULTS
Copyright American Society of Nuclear Cardiology

## Slide 6
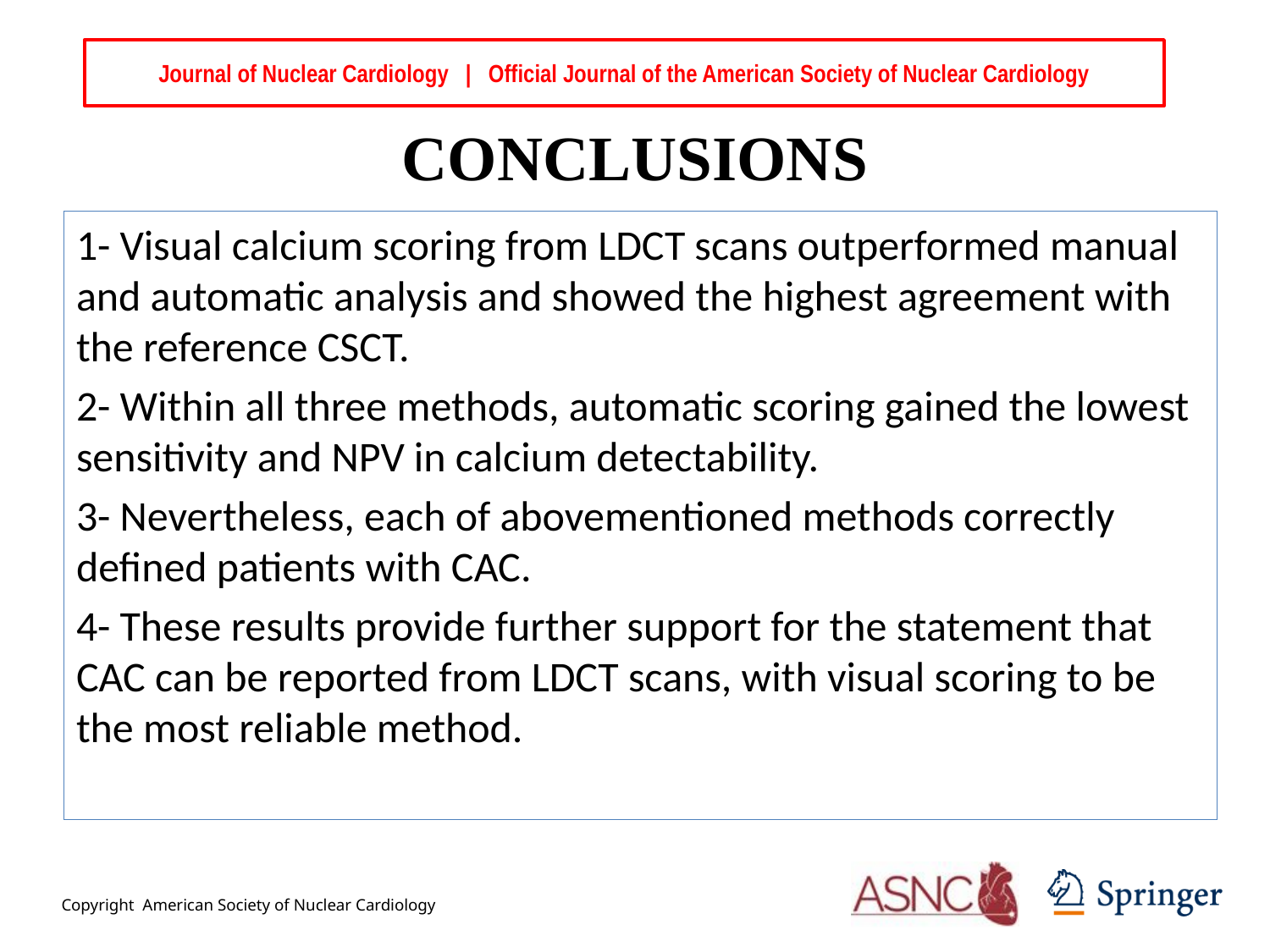

Journal of Nuclear Cardiology | Official Journal of the American Society of Nuclear Cardiology
# CONCLUSIONS
1- Visual calcium scoring from LDCT scans outperformed manual and automatic analysis and showed the highest agreement with the reference CSCT.
2- Within all three methods, automatic scoring gained the lowest sensitivity and NPV in calcium detectability.
3- Nevertheless, each of abovementioned methods correctly defined patients with CAC.
4- These results provide further support for the statement that CAC can be reported from LDCT scans, with visual scoring to be the most reliable method.
Copyright American Society of Nuclear Cardiology
